# Spatio-temporal variations of emerging sites infested with schistosome-transmitting *Oncomelania hupensis* in Hunan Province, China, 1949–2016

**DOI:** 10.1186/s13071-020-04526-z

**Published:** 2021-01-06

**Authors:** Shengming Li, Ying Shi, Weicheng Deng, Guanghui Ren, Hongbin He, Benjiao Hu, Chunlin Li, Na Zhang, Yingyan Zheng, Yingjian Wang, Shurong Dong, Yue Chen, Qingwu Jiang, Yibiao Zhou

**Affiliations:** 1 Hunan Institute for Schistosomiasis Control, Yueyang, Hunan China; 2 Fudan University School of Public Health, Building 8, 130 Dong’an Road, Xuhui District, Shanghai, 200032 China; 3 Key Laboratory of Public Health Safety, Ministry of Education, Fudan University, Building 8, 130 Dong’an Road, Xuhui District, Shanghai, 200032 China; 4 Fudan University Center for Tropical Disease Research, Building 8, 130 Dong’an Road, Xuhui District, Shanghai, 200032 China; 5 School of Epidemiology and Public Health, Faculty of Medicine, University of Ottawa, 600 Peter Morand Crescent, Ottawa, Ontario K1G 5Z3 Canada

**Keywords:** *Schistosomiasis japonica*, *Oncomelania hupensis*, Moran’s I, LISA, Space-time scan statistics, Durations of snail habitats

## Abstract

**Background:**

Constant emerging sites infested with *Oncomelania hupensis* (*O. hupensis)* impede the goal realization of eliminating schistosomiasis. The study assessed the spatial and temporal distributions of new *Oncomelania* snail habitats in Hunan Province from 1949 to 2016.

**Methods:**

We used the data from annual snail surveys throughout Hunan Province for the period from 1949 to 2016. Global Moran’s *I*, Anselin local Moran’s *I* statistics (LISA) and a retrospective space-time permutation model were applied to determine the spatial and temporal distributions of emerging snail-infested sites.

**Results:**

There were newly discovered snail-infested sites almost every year in 1949–2016, except for the years of 1993, 2009 and 2012. The number of emerging sites varied significantly in the five time periods (1949–1954, 1955–1976, 1977–1986, 1986–2003 and 2004–2016) (*H* = 25.35, *p* < 0.05). The emerging sites lasted 37.52 years in marshlands, 30.04 years in hills and 24.63 at inner embankments on average, with the values of Global Moran’s *I* being 0.52, 0.49 and 0.44, respectively. High-value spatial clusters (HH) were mainly concentrated along the Lishui River and in Xiangyin County. There were four marshland clusters, two hill clusters and three inner embankment clusters after 1976.

**Conclusions:**

Lower reaches of the Lishui River and the Dongting Lake estuary were the high-risk regions for new *Oncomelania* snail habitats with long durations. Snail surveillance should be strengthened at stubborn snail-infested sites at the inner embankments. Grazing prohibition in snail-infested grasslands should be a focus in marshlands. The management of bovines in Xiangyin County is of great importance. 
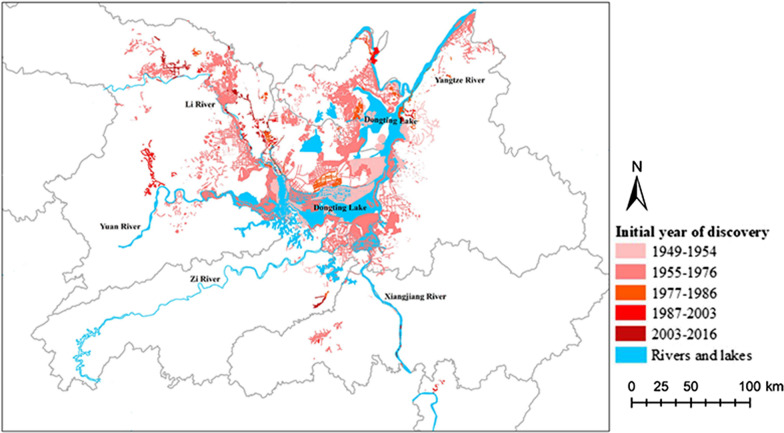

## Background

*Schistosomiasis japonica* is a chronic and debilitating disease that also exacerbates poverty [1]. The World Health Organization (WHO) estimated that approximately 200,000 deaths were caused by schistosomiasis and 290.8 million people required preventive treatment for schistosomiasis worldwide in 2018 [1–3]. Schistosomiasis caused a loss of nearly 2 million disability-adjusted life years in 2016 [4].

In China, schistosomiasis is caused by trematode blood fluke *Schistosoma japonicum (S. japonicum)*. As the sole intermediate host, *Oncomelania hupensis (O. hupensis)* plays a critical role in the transmission of schistosomiasis japonica [5], and the geographic distribution of snails determines the occurrence of schistosomiasis [6]. The schistosomiasis-endemic areas in China are classified into three types according to eco-geographical characteristics: (i) marshland and lake regions, (ii) mountainous and hilly regions and (iii) plain regions with waterway networks [7]. Hunan Province retains the first two types, and the marshland and lake-endemic area is further divided into subtypes (marshland and inner embankment) according to the variability of hydrology [8]. Dongting Lake and its tributaries, lying in the northeast of Hunan Province, dominate the marshland and lake regions outside the embankments. During the spring and summer flooding season, the lake and rivers inundate a large area outside the embankments while in the winter they shrink considerably [9]. The characteristic of ‘marshlands in winter and water in summer’ is ideal for the survival of *O. hupensis*. The measures of returning farmland have enlarged the marshland as well as the snail area [9].

WHO has set the target of ‘morbidity control’ by 2020, which aims to reduce the prevalence of heavy-intensity infection to ≤ 5% among school-aged children [10], and a recommended strategy is to control the freshwater snails that transmit schistosome parasites [11]. Snail control measures are constantly conducted in Hunan Province, but emerging sites infested with *O. hupensis* occur frequently [12]. *O. hupensis* were eliminated in most original places in the 1950s, but they could immigrate from the original sites to places with a more suitable environment [13]. Because of enlarged areas of marshland with widely distributed and complicated habitats of snails, the past experience of large-scale reclamation is no longer suitable for the purpose of eliminating snails [8]. In our study, a snail habitat is defined as the area where the snails can survive and reproduce. It is important to identify and monitor new snail habitats to effectively control schistosomiasis [14]. This study aimed to investigate the spatio-temporal patterns of the new *Oncomelania* snail habitats in Hunan Province from 1949 to 2016.

## Materials and methods

### Study sites

**Fig. 1:**
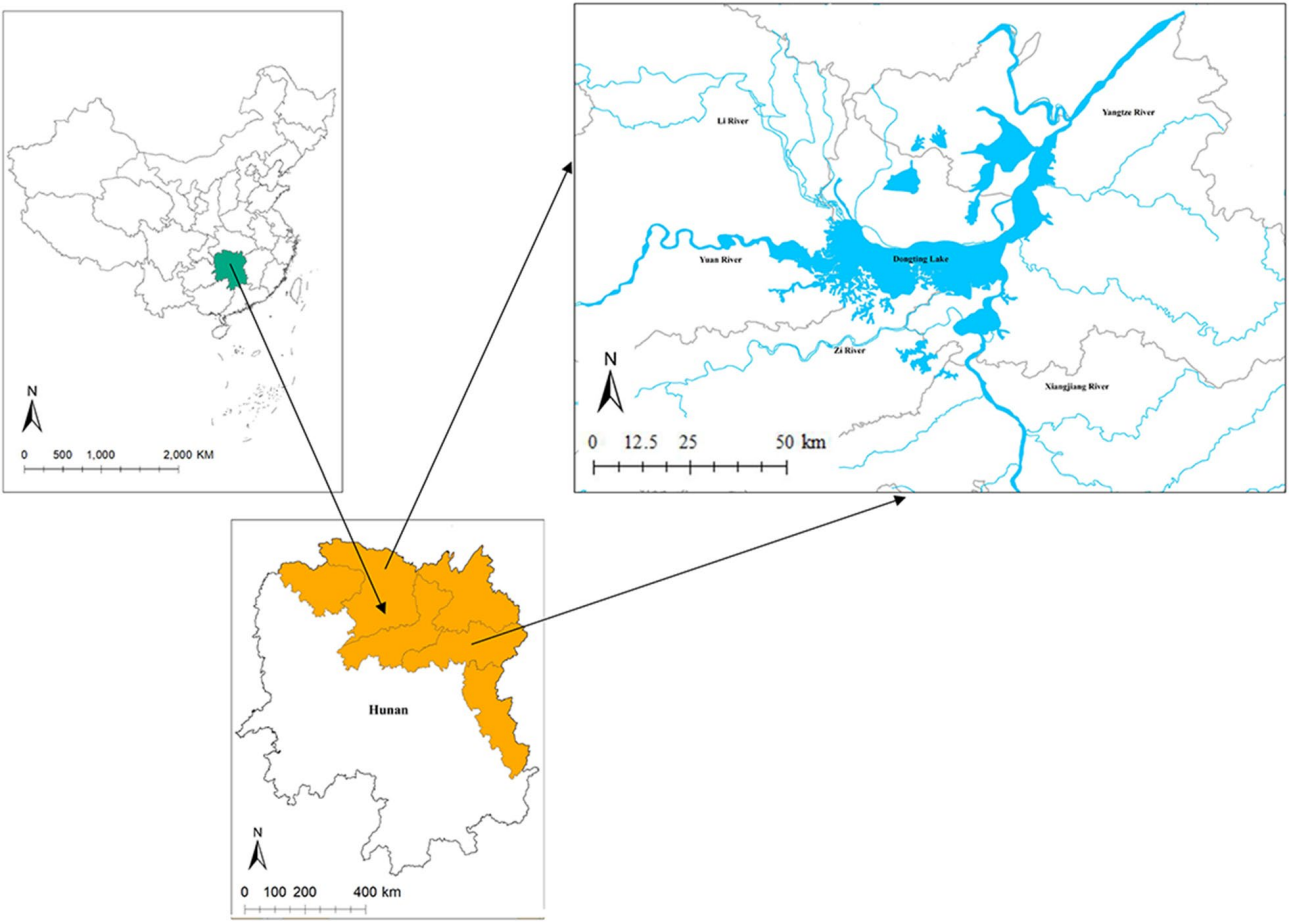
Geographical location of the study region in Hunan Province of China

The study was conducted in Hunan Province, China (Fig. 1), which is located on the south bank of the Yangtze River, situated between 108°47′–114°16′ east longitude and 24°37′–30°08′ north latitude [15]. As a humid subtropical monsoon climate zone, winter is short, cool and damp, and summer is hot and humid, with an annual rainfall of 1300–1800 mm. The temperature is about 3 to 8 ℃ in January and 27 to 30 °C in July [16]. Hunan has 45.9% of the total snail-infected area (3.85 billion square meters) in the country. Dongting Lake lies in the northeast of Hunan Province, connecting water with the lower branch of the Yangtze River. The characteristic of ‘marshlands in winter and water in summer’ is ideal for the survival of *O. hupensis*. Four rivers (Xiang, Zi, Yuan and Li) converge into the Yangtze River at Dongting Lake [16]. An inner embankment is defined as an area close to the lake but inside the embankment. Foreign snails can effortlessly migrate from marshlands to inner embankments. Mountainous habitats of Hunan provinces account for 17% of all snail mountainous habitats in The People’s Republic of China [17]. In the mountainous and hilly regions, the snails are mainly distributed in ditches and rice paddies [18].

### Materials

The annual snail surveys carried out throughout Hunan Province from 1949 to 2016 contain information on the location, area, and first and last years of emerging snail-infested sites. An emerging snail-infested site was defined as a new site infested with the snails in a particular year. The first year of an emerging site was defined as the earliest year to identify living *O. hupensis* in the site. The last year of an emerging site was the year that the snails disappeared from the site. The duration of an emerging snail-infected site was the difference between the last year and the first year of the emerging site.

The annual snail surveys are performed using a traditional random equidistant frame survey method combined with random quadrant sampling (0.11 m^2^-sized frames) directly. The frames are set 20 × 20 m apart when the area outside the embankments is < 200,000 km^2^ or 50 × 30 m apart when the area is > 200,000 km^2^. For the inner embankments, the frames are set at 5 m × 10 m apart. All captured snails are examined to identify living ones by microscope after crushability. The latitude and longitude of snail-infested sites were obtained by a handheld global positioning system (GPS). The distribution maps were drawn by Google Earth via key inflexion points.

### Quality control

A professional control system for snail field surveys was established after the People's Republic of China (PRC) was established in 1949 [6] and provides valuable historical records about emerging snail habitats. The survey results are published every year [19, 20]. The quality control for each snail survey includes professional training of investigators, supervision by provincial officials and reexamination of snail samples [21].

### Descriptive analysis

A database was established using Microsoft Excel 2016 (Microsoft Inc. Washington, DC, USA). Descriptive analysis was performed using the statistical software package R 3.6.1 (AT&T Bell Laboratories, New Zealand). In line with the national schistosomiasis control programs in China [22], we examined the differences in the spatio-temporal variations of emerging snail-infested sites among five time periods: preparation period (1949–1954), mass campaign period (1955–1976), achievement consolidation period (1977–1986), morbidity control period (1986–2003) and comprehensive strategy period (2004–2016). Kruskal-Wallis tests were performed to determine the difference in the average number of snail-infested sites among five time periods. A coordinated Nemenyi pairwise comparison was made for the duration of emerging snail-infected sites among three geographical categories (marshlands, inner embankments and hills).

### Spatial autocorrelation analysis

Global Moran’s *I* was applied to detect spatial autocorrelation for the durations of emerging snail-infected sites among the three categories (marshlands, inner embankments and hills). A row standardization (a technique for adjusting the weights in a spatial weight matrix) was conducted. The method of inverse distance was applied to determine the spatial relationship. The value of the Moran's *I* ranges between −1 (maximum negative association) and 1 (maximum positive association), with a zero value indicating a random spatial pattern [23]. A *Z*-score > 1.96 or < $-1.96$ and a two-sided *p* value < 0.05 were considered to be statistically significant.

Local indicators of spatial association (LISA) were used in the present study to detect the actual location of significant spatial clusters and outliers [24]. It was adopted to explore significant hotspots (HH: high-value spatial clusters with long durations), coldspots (LL: low-value spatial clusters with short durations) and outliers (HL: high values surrounded with low values, LH: low values surrounded with high values) of durations by calculating local Moran’s *I* index. A high positive *z* score indicated that the surroundings had spatial clusters and a low negative *z* score indicated the presence of spatial outliers [24]. The LISA map was based on 999 permutations, and features with a *p*-value < 0.05 were considered statistically significant [25]. All spatial processing and mapping were carried out in ArcGIS 10.0 (ESRI, Inc. Redlands, CA, USA).

### Spatio-temporal analysis

The data of each site with longitude, latitude and initial year of snail discovery were imported into SaTScan 9.6 (Kulldorff & Information Management Services, Inc., Bethesda, MD, USA) for retrospective spatiotemporal aggregation analysis. The space-time permutation model requires only case data. The number of attribute values in the time-space window complied with the hypergeometric distribution, and the expected incidence *μ*(*z*) of each scanning window *Z* was calculated according to the characteristics of the hypergeometric distribution [26]:

$$\mu \left( Z \right) = \mathop \sum \limits_{{\left( {Z,d} \right) \in Z}} \left[ {\frac{1}{{n_{G} }}\left( {\mathop \sum \limits_{Z} n_{Zd} } \right)\left( {\mathop \sum \limits_{d} n_{Zd} } \right)} \right]$$

The *p*-value was generated using the Monte Carlo hypothesis testing method with 999 simulations, and the cut-off point was set at 0.05 [27]. Maximum spatial cluster size was limited to 50% of the population at risk, and the minimum temporal cluster size was 1 year.

## Results

### Time and space distributions of emerging *O. hupensis* snail-infested sites

**Fig. 2:**
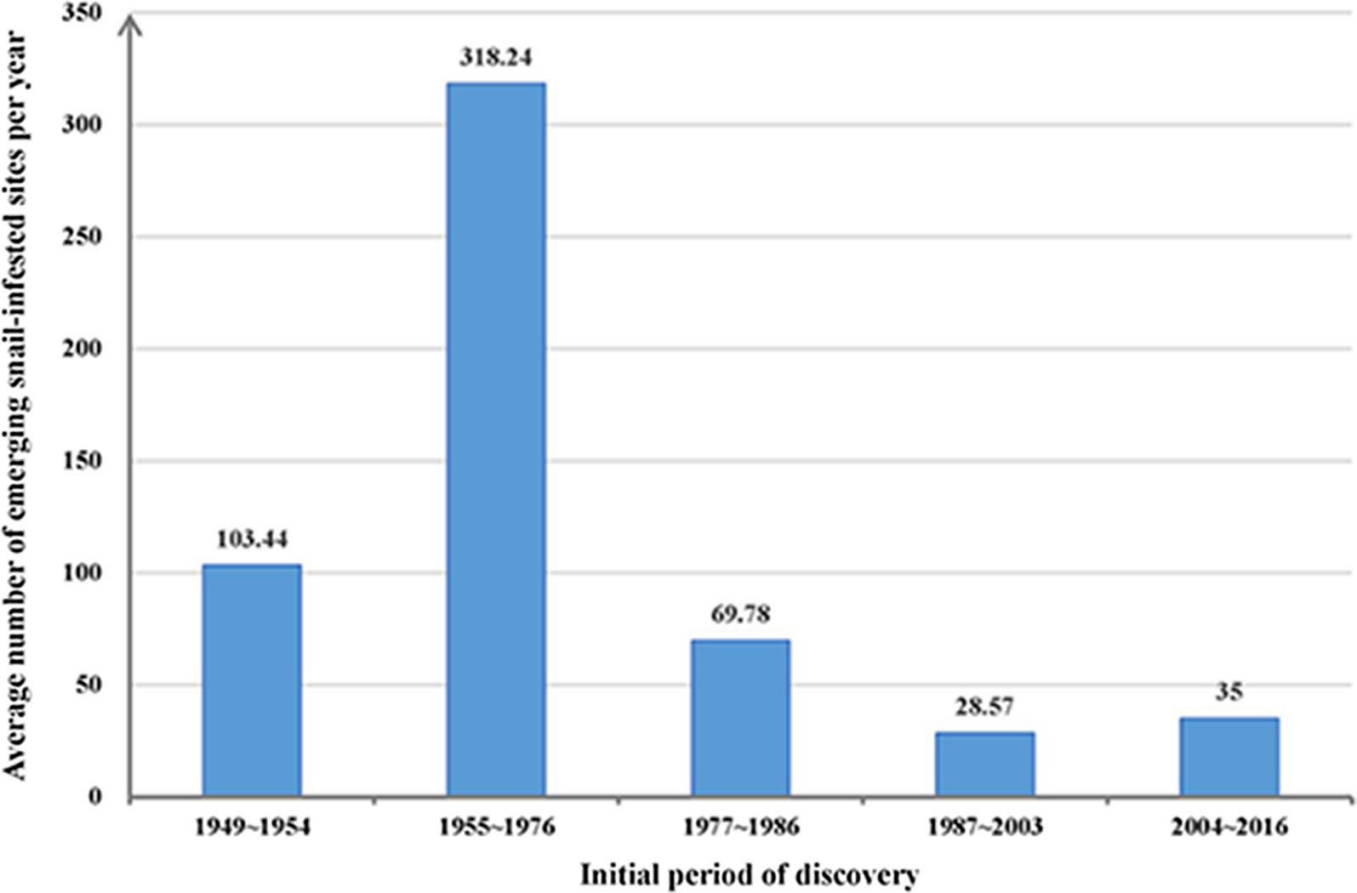
Number of emerging snail-infested sites in five periods (1949–1954, 1955–1976, 1977–1986, 1986–2003 and 2004–2016), Hunan Province

**Fig. 3:**
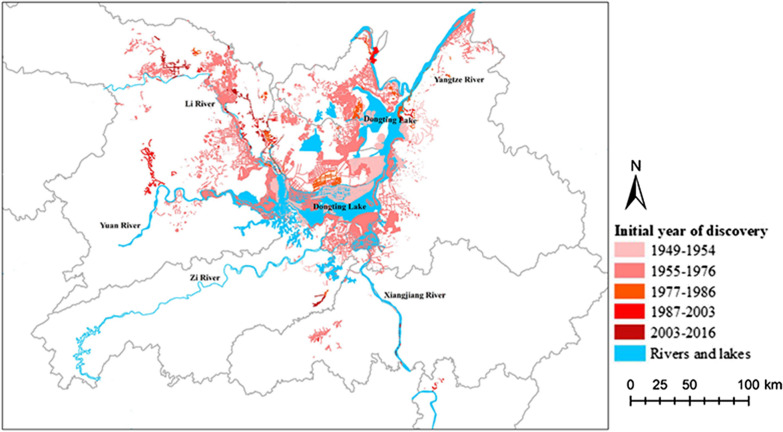
Distribution of emerging snail-infested sites in Hunan Province in different time periods. The site with the lightest red part was discovered in 1949–1954, and so on. The darkest red part was for 2003–2016

Figure 2 shows the number of emerging snail-infested sites in the five periods (Kruskal-Wallis test: *H* = 25.35, *P* < 0.05). Compared with the preparation period (1949–1954), the number of emerging sites increased up to 318.24 per year in the second period. Then, the number declined by 78.07% (248.46 per year) in the third period (1977–1986) and 91.02% (289.67 per year) in the fourth period (1987–2003) compared with the second period. However, the number increased 22.51% (6.43 per year) during the fifth period (2004–2016) compared with the fourth period. Geographically, the emerging snail-infested sites in the preparation period (1949–1954) clustered mostly at the junction of East Dongting Lake and South Dongting Lake with a total area of 9.8 × 10^9^ km^2^. In the second period, the emerging sites showed a diffuse distribution with an infected area of 4.34 times larger (5.3 × 10^10^ km^2^) compared with the first period. In the third period, > 600 snail-infested sites were discovered, but the infected area decreased by 68.02% compared with the second period. Although the number of emerging sites was the lowest in the fourth period, the infected area was 30.83% larger compared with the third period. The emerging sites dispersed mainly along Lishui River in the fifth period. Average infested area was 30.32% smaller than that in the fourth period. The space distribution of emerging *O. hupensis* snail-infested sites in the five periods is shown in Fig. 3.

### Description and spatial autocorrection of durations

Mean and median durations of emerging snail-infected sites were 29.68 years and 23 years overall and varied across geographical locations. The time span of the sites was 67 years both inside and outside the embankments, but was 70 years in hilly areas. Mean duration of the emerging sites was longer in regions outside embankments than that in hilly areas (37.52 vs. 30.04 years) and was the shortest in regions inside embankments (24.63 years) (Kruskal-Wallis test: *H* = 471.63, *P* < 0.05). Global Moran’s *I* showed a significant positive spatial autocorrelation for the durations of emerging snail-infected sites in all three schistosomiasis-endemic categories in Hunan Province (Moran’s *I* > 0.4, *P* < 0.01) (Table 1).

**Table 1:** Value of Moran’s *I* and its statistical significance for snail habitats in Hunan Province

Category	Moran’s *I*	Expected index	Variance	Z Score	*P-*value
Inner embankments	0.44	−0.00067	0.000067	53.17	< 0.01
Marshlands	0.52	−0.000917	0.000358	27.30	< 0.01
Hills	0.49	−0.000872	0.000054	66.38	< 0.01

**Fig. 4:**
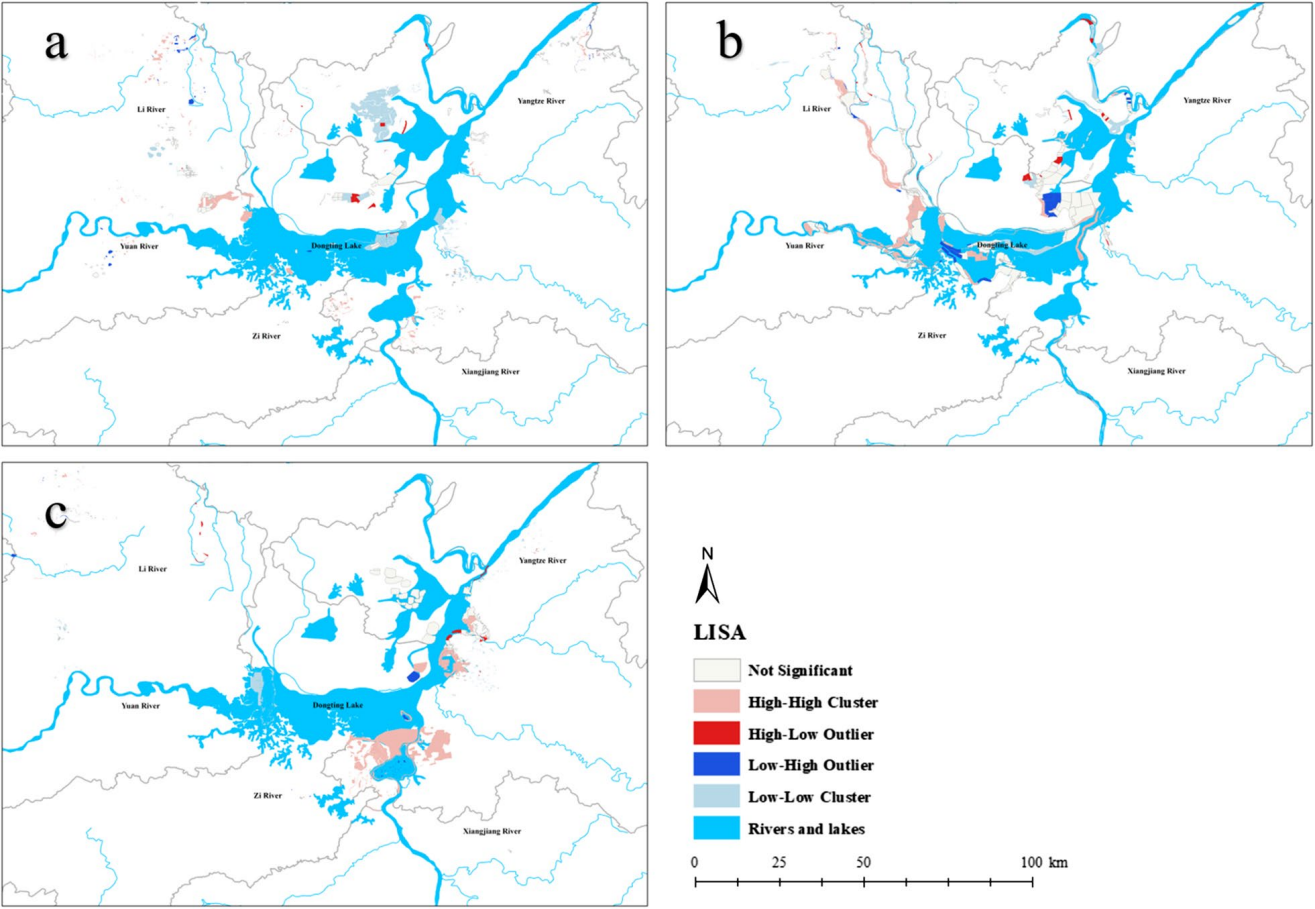
Anselin local Moran's *I* clustering of durations among **a** inner embankments, **b** in marshlands and **c** in hills. The pink parts indicate HH clusters where snails were in aggregation. The red and blue parts indicate HL outliers and LH outliers where snails may spread

Significant clustering (HH, LL, HL and LH) is shown in Fig. 4. In all types of emerging snail-infested aread, there were many more HH clusters than HL and LH outliers. In the sites confirmed positive by the annual snail census, there were more HH clusters than LL clusters, indicating more frequent clustering for snail-infected sites with a longer duration than those with a shorter duration. HL villages had the fewest in the three categories.

In the area of inner embankments, HH clusters were found in the north-western part of Dongting Lake. LL clusters at inner embankments were observed in the west of East Dongting. HH clusters in marshlands were mainly concentrated along Lishui River and Yuanshui River. LH outliers were mainly distributed in the center of Dongting Lake and the corner between East Dongting and South Dongting. In the area of hills, HH clusters were mainly distributed in Xiangyin County.

### SaTScan clusters of emerging snail-infested sites

#### Inner embankments

**Fig. 5:**
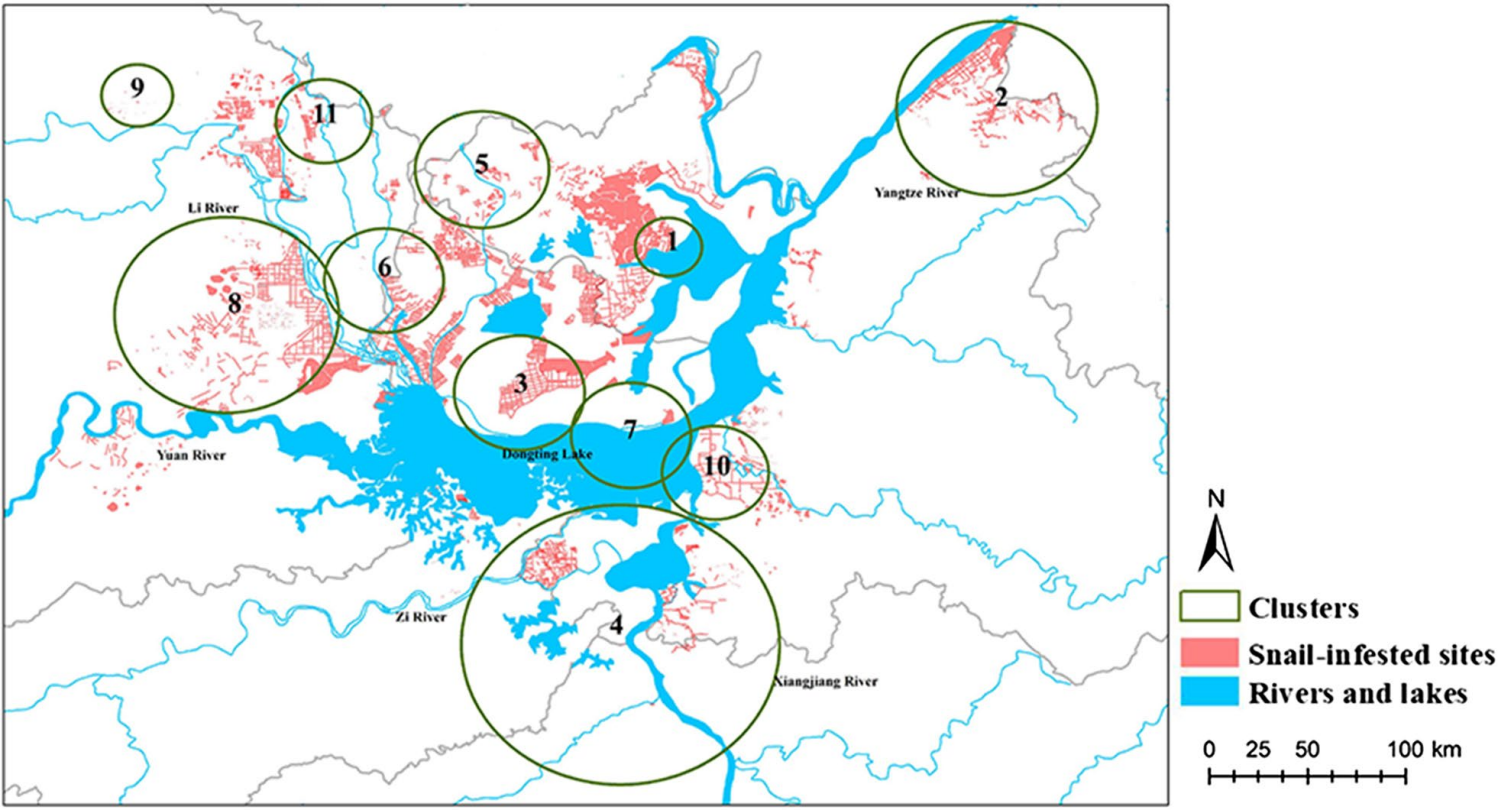
Spatial distribution of clusters of the emerging snail-infested sites of the inner embankments. Snails were spatially concentrated in clusters. The tighter they gathered, the smaller the cluster was

A total of 4475 emerging snail-infested sites were included in the space-time analysis to describe the spatial distribution of inner embankments and to investigate the presence of high-density clusters. Eleven statistically significant clusters (*P* < 0.05) were identified by the space-time permutation model (Table 2, Fig. 5).

**Table 2:** Clusters of emerging snail-infested sites inside embankments from 1949 to 2016

Clusters	Radius (km^2^)	Time frame	Duration of cluster (years)	Observed number	Expected number	Observed/expected	*P-*value
1	6.89	1978–1980	3	227	16.70	13.59	< 0.01
2	20.67	1956	1	760	263.98	2.88	< 0.01
3	13.53	1970	1	136	5.51	24.67	< 0.01
4	33.17	1963–1965	3	162	18.77	8.63	< 0.01
5	13.81	1953–1955	3	162	18.83	8.60	< 0.01
6	12.34	1984–2011	28	88	4.36	20.19	< 0.01
7	12.43	1952–1953	2	82	4.31	19.04	< 0.01
8	23.20	1960–1968	9	412	149.60	2.75	< 0.01
9	7.33	2006–2015	10	44	0.95	46.26	< 0.01
10	11.07	1958	1	84	7.66	10.97	< 0.01
11	9.91	1961–1962	2	119	19.97	5.96	< 0.01

Cluster 6 (1984–2011) with a radius of 12.34 km^2^, located in the northwest of Dongting Lake, had the longest duration of 28 years, and the observed to expected ratio (O/E ratio) for the number of infected sites was 20.19. Cluster 9 (2006–2015), located in the northwest of cluster 6 with a radius of 7.33 km^2^, had the second longest duration of 10 years, and the O/E ratio was 46.26, the largest among all clusters.

### Marshlands

**Fig. 6:**
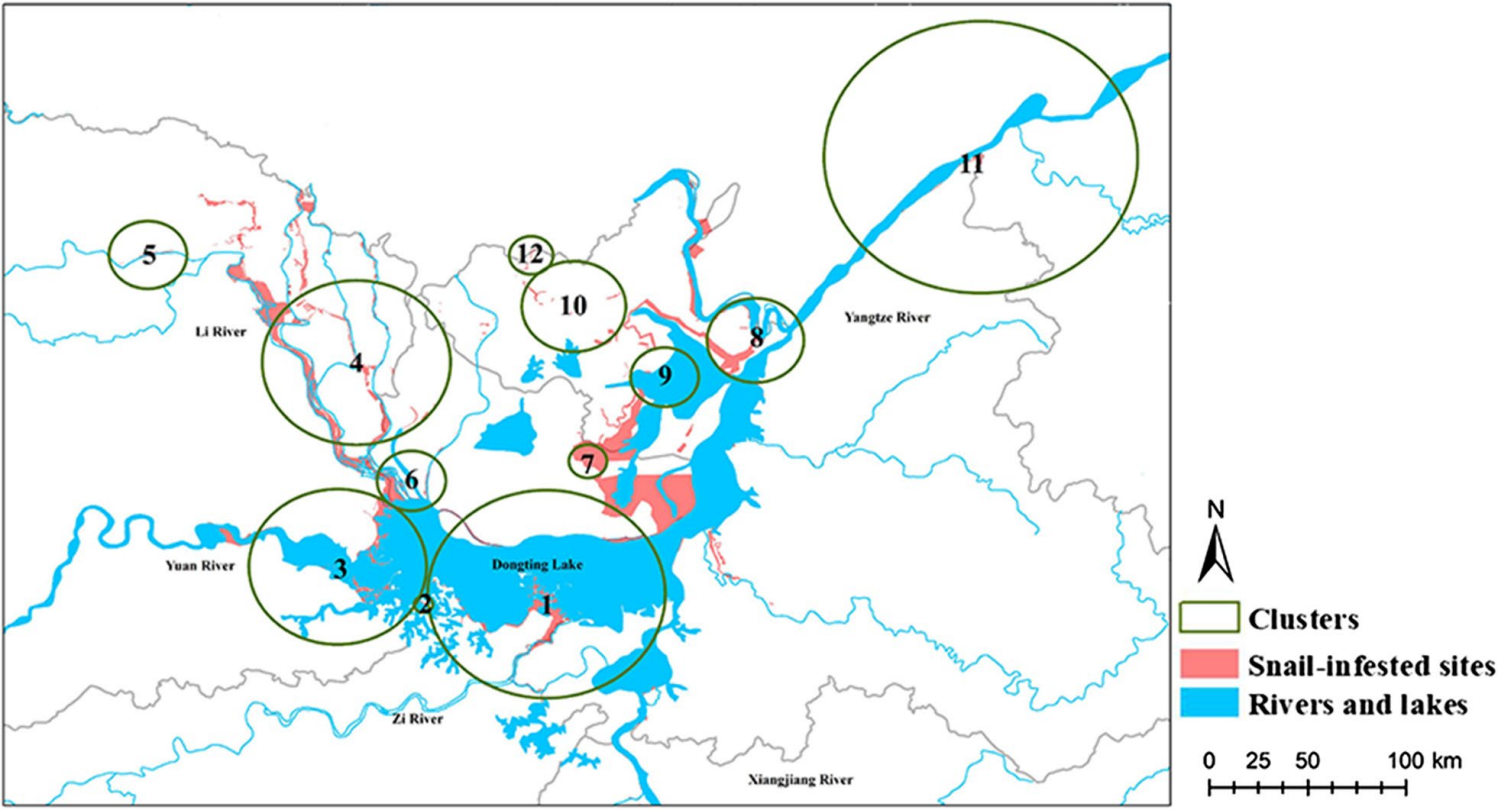
Spatial distribution of clusters of the emerging snail-infested sites in marshlands. They mainly gathered along rivers

Among the 1101 emerging snail-infested sites in outside embankments included in the study, there were 12 significant clusters (Table 3, Fig. 6). Only Clusters 4 and 8 existed > 2 years, with durations of 14 and 2 years, respectively. Cluster 4 (2003–2013), located in the northwest of Dongting Lake, had a radius of 20.56 km^2^. Cluster 5 (2015) was the most recent one, but only existed for 1 year. Cluster 5, located in the northwest of Cluster 4, had a radius of 8.48 km^2^ and an O/E ratio for the number of infected sites of 33.82.

**Table 3:** Clusters of emerging snail-infested sites outside embankments from 1949 to 2016

Clusters	Radius (km^2^)	Time frame	Duration of cluster (Years)	Observed number	Expected number	Observed/expected	*P*-value
1	26.07	1953	1	98	16.29	6.01	< 0.01
2	2.05	1974	1	28	0.84	33.15	< 0.01
3	19.46	1956	1	119	34.88	3.41	< 0.01
4	20.56	2003–2013	14	50	7.14	7.00	< 0.01
5	8.48	2015	1	20	0.59	33.82	< 0.01
6	7.52	1984	1	23	1.14	20.10	< 0.01
7	4.16	1959	1	16	0.36	44.48	< 0.01
8	10.56	1954–1955	2	19	1.12	17.01	< 0.01
9	7.42	1950	1	12	0.32	37.53	< 0.01
10	11.34	1962	1	15	0.79	19.05	< 0.01
11	34.09	1956	1	44	12.79	3.44	< 0.01
12	4.77	1996	1	6	0.054	110.10	< 0.01

### Hills

**Fig. 7:**
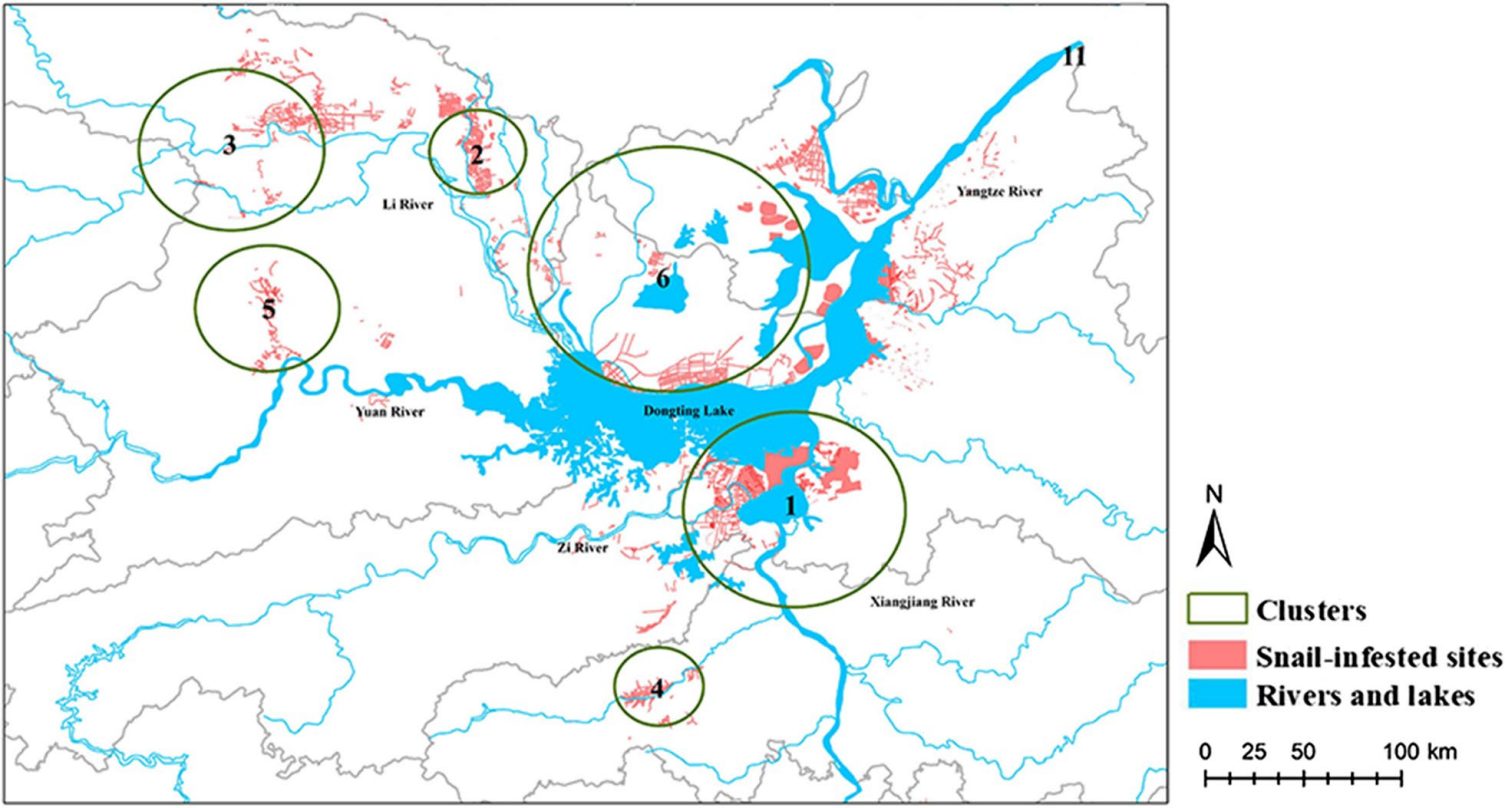
Spatial distribution of clusters of the emerging snail-infested sites in hills. They mainly gathered around streams, away from marshlands and lake regions

There were six significant clusters in hills (Table 4). Cluster 5 (1996–1998) existed in the fourth period and was located in the northwest of Dongting Lake, with an O/E ratio for the number of infected sites of 15.65. Cluster 6 (1970–1984) had a largest radius (34.57 km^2^) and the longest duration (15 years) but the fewest observed sites among all clusters. It appeared in the third period in the north of Dongting Lake, with an O/E ratio of 8.69. Cluster 1 had the largest number of infected sites and a radius of 27.52 km^2^. Other clusters only appeared in the second period (Fig. 7).

**Table 4:** Cluster of emerging snail-infested sites in hills from 1949 to 2016

Clusters	Radius (km^2^)	Time frame	Duration of cluster (years)	Observed number	Expected number	Observed/expected	*P*-value
1	27.52	1960–1965	6	587	142.33	4.12	< 0.01
2	11.84	1958	1	298	51.45	5.79	< 0.01
3	22.75	1955–1956	2	406	100.51	4.04	< 0.01
4	11.07	1957	1	104	3.41	30.54	< 0.01
5	17.78	1996–1998	3	136	8.69	15.65	< 0.01
6	34.57	1970–1984	15	135	15.54	8.69	< 0.01

### Combination of LISA and SaTScan

**Fig. 8:**
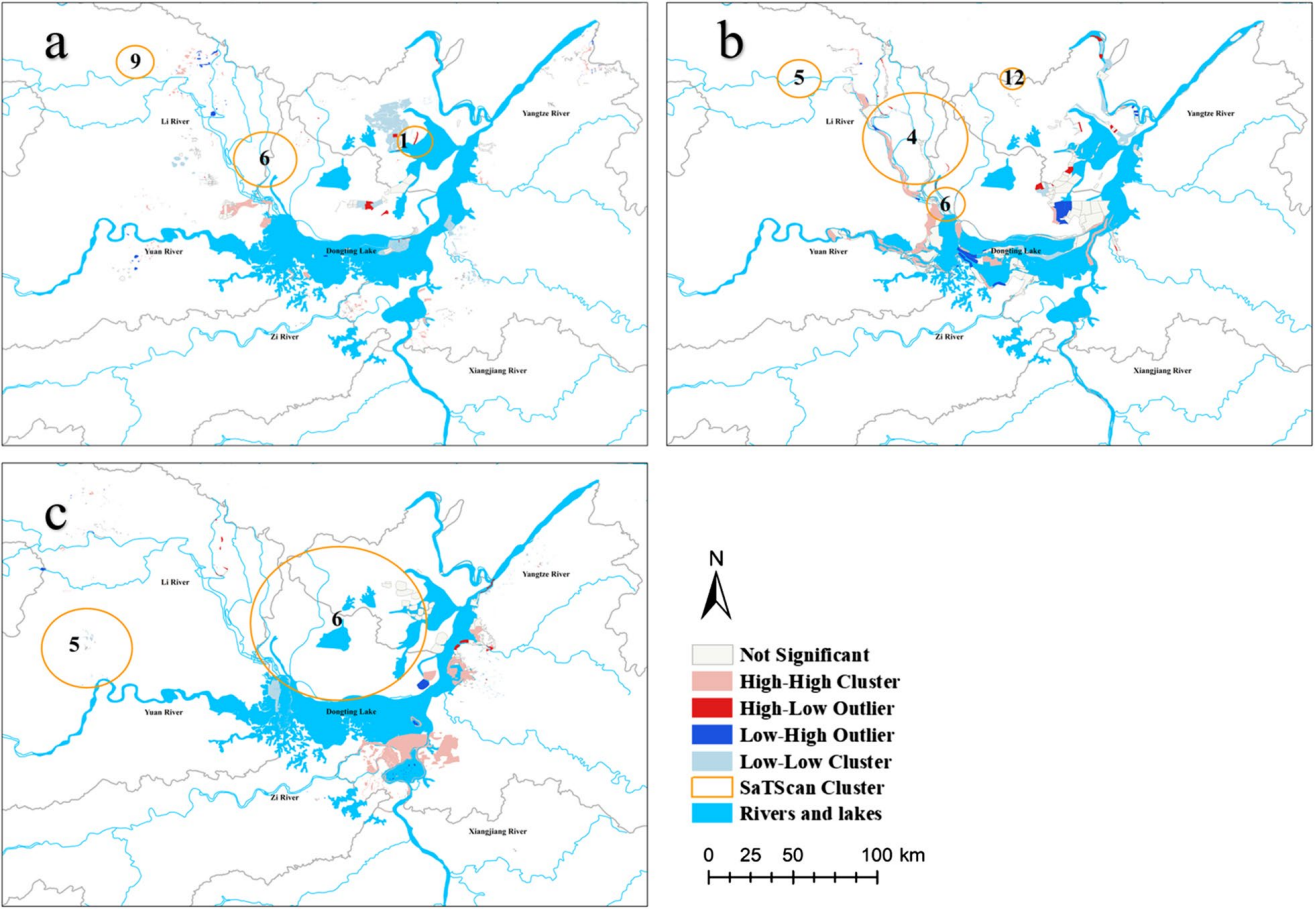
Combination of LISA and SaTScan: **a** inner embankments, **b** in marshlands and **c** in hills. Hotpots of LISA and clusters with long durations (> 1 year) were combined in maps, and the overlapped regions were at high risk

The clusters after 1976 were included, and the combination of LISA and SaTScan screened the most significant zones (Fig. 8). In the categories of inner embankments, HH clusters did not overlap with Clusters 6 and 9. Cluster 1 covered the HL outlier. In the region of marshlands, Clusters 4 and 6 covered most of the HH clusters. However, Clusters 5 and 12 covered the zone with no significance in LISA. In the hills, Cluster 5 covered the LL clusters, and Cluster 6 covered the area with no significance in LISA.

## Discussion

Annual snail surveys and monitoring of suspected snail-infested environments has been successively conducted since 1949 in Hunan Province. Although snail control has always been ongoing, new habitats of *Oncomelania* snails have emerged frequently. Earlier epidemiological surveys during 1950–1953 [15] and later [28] discovered a large number of new snail habitats in Hunan Province. Although an elimination strategy was implemented and resulted in a great decrease of the snail-infected area during 1956–1976 [29], there have been many new habitats of *Oncomelania* snails since 1977. Most of the clusters in SaTScan existed in the 1950s and 1960s, and the clusters after 1976 reflected the emerging snail-infested sites. These emerging snail habitats mainly came from the diffusion of the original sites. Genetic analysis demonstrated that snails in emerging habitats were introduced through migration from original sites [30]. From 1977 to 2016, emerging snail-infected sites were discovered in most years, with the exception of 1993, 2009 and 2012, indicating that snail control is a tough mission. Despite the prevalence of schistosomiasis had been decreasing, the number of emerging habitats of *O. hupensis* continued to increase from 2003 to 2016.

The results from the global spatial autocorrelation analysis indicated spatial heterogeneity in the durations of emerging snail habitats for all the categories of regions. The marshlands are noteworthy for its overwhelming proportion (99.64%) [31]. In marshlands, the Lishui River’s upstream had fewer HH clusters than the middle and lower reaches. The fast flows in the upper reaches threaten the survival of snails [32]. The reasons for long durations in the lower reaches could be bend flow and deposited silt, which are suitable for snails’ growth and migration [32]. The anomaly of the multi-year average runoff has increased in the four rivers since the operation of the Three Gorges Dam, leading to vast grass-covered marshlands and areas for snails [33, 34]. A previous study also showed that the dominant effect of vegetation was reinforced in the north of the Lishui River plain [35], so that the vegetable coverage and specific vegetation types should be considered in snail control programs in the region. The practice of planting fast-growing and adapting trees leads to changes in soil temperature, humidity, pH values and vegetation density, which can make the land less susceptible to *O. hupensis* [36]. Other HH clusters to be concerned are in the highlands of the Xiangjiang River estuary, mainly in Xiangyin County. The bovine population in Xiangyin County ranks first in Hunan Province [31], and free-range of cattle may contributes to the spread of schistosomiasis. The combination of LISA and SaTScan showed that the lower reaches of Lishui River and Dongting Lake estuary were at high risk. Since LISA focuses on geographical connections while SaTScan on both space and time, the results are not entirely consistent.

By comparing the results of Local Moran’s *I* statistics and SaTScan, we found overlaps between HH areas in LISA and long-duration circles in SaTScan. The two spatial methods provided consistent results in detecting clusters. After the floods in 2014, 1983 and 1995, respectively, Clusters 5 (2015), 6 (1984) and 12 (1996) emerged subsequently [37, 38]. Flooding and unstable water levels as well as vast marshland regions are likely the main factors leading to the long existence of emerging sites in marshlands. The emerging sites were mostly in the basins of the four rivers, which are frequently inundated by flooding. Snails can be adsorbed on floating debris and spread with water flows [39] or directly spread with the flood water when embankments collapse [39, 40]. Flooding can accelerate the siltation and the formation of new marshland, which is conducive to the expansion of emerging snail habitats [39], and increase the emerging snail-infested sites over the next several years [41]. Dongting Lake is still at high risk for severe flooding and water logging in dyke-enclosed places after the full operation of the Three Gorges Dam [33, 42]. It is difficult to eliminate snails in marshlands by molluscicide or environmental modification. Although great efforts have been made toward snail elimination, new snail-infested sites appear frequently, which is a big challenge for snail control program development. Since the water level is difficult to control, grazing prohibition in snail-infested grasslands should be the first priority [36].

The durations of emerging snail habitats at inner embankments were longer than those in marshlands after 1976. Emerging snail-infested sites at inner embankments were stubborn ones, and those in marshlands were relatively transient. The clusters were not likely to be totally independent. The inner embankment area is isolated from the marshland by an embankment, but is connected to the marshland by sluice gates. There has been effective snail control management inside the embankments [43]. Once snails spread into the inner embankments with flood water, it may take several years to remove them completely. A thick riverside settlement and the redirection of available water for irrigation systems may also contribute to a long duration of snail-infected sites [44]. The geographical environment of the endemic areas does not change fundamentally, and the crisscross ditches and climate are suitable for snail survival [45]. The surveillance around old stubborn snail-infested areas and environmental modification should be strengthened at the inner embankments.

There are also hilly snail-infested regions in Hunan. It is difficult to eradicate snails in hills because of their complex environment such as loose soil and stony land [1, 45]. Geographically, most of hilly snail-infested regions are away from the marshland and lake regions and are rarely affected by flooding. The inner embankment area is isolated from the marshland by an embankment, but is connected to the marshland by sluice gates. The inner embankment is rarely affected by flooding, but the marshland is.

A limitation of the study is the lack of historical snail density information, which hinders a further quantitative analysis. Environmental factors need to be further explored for emerging snail-infected sites in different geographical regions. Given the different spatial analysis units, the clusters detected were not exactly in the same areas. For example, the HH clusters of inner embankments did not overlap with Clusters 6 and 9.

## Conclusions

The lower reaches of Lishui River and the Dongting Lake estuary are high-risk regions for new *Oncomelania* snail habitats with long durations. The snail surveillance should be strengthened around the stubborn snail-infested sites at the inner embankments. The grazing prohibition in snail-infested grasslands should be a focus in marshlands. The management of bovines in hills is of great importance, especially in Xiangyin County.

## Data Availability

The datasets used and/or analyzed during the current study are available from the corresponding author on reasonable request.

## Abbreviations

WHO: World Health Organization
S. japonicum: Schistosoma japonicum
LISA: Local Indicators of Spatial Association
O. hupensis: Oncomelania hupensis
GPS: Global Positioning System
PRC: People's Republic of China
HH: High-value spatial clusters
LL: Low-value spatial clusters
HL: High values surrounded with low values
LH: Low values surrounded with high values
